# Supplementary material for: Comparative multiomics analysis of cell physiological state after culture in a basket bioreactor
Source: Sci Rep. 2022 Nov 23;12:20161. doi: 10.1038/s41598-022-24687-4 (PMC9686226; doi:10.1038/s41598-022-24687-4)
Supplement: Supplementary file 1 — Supplementary Information 1. [file 41598_2022_24687_MOESM1_ESM.zip › raw data/Transcriptomic raw data/6.Enrichment/1.GO/TE1vsTC1/ALL/TE1vsTC1.all_GObar_bp.pdf]

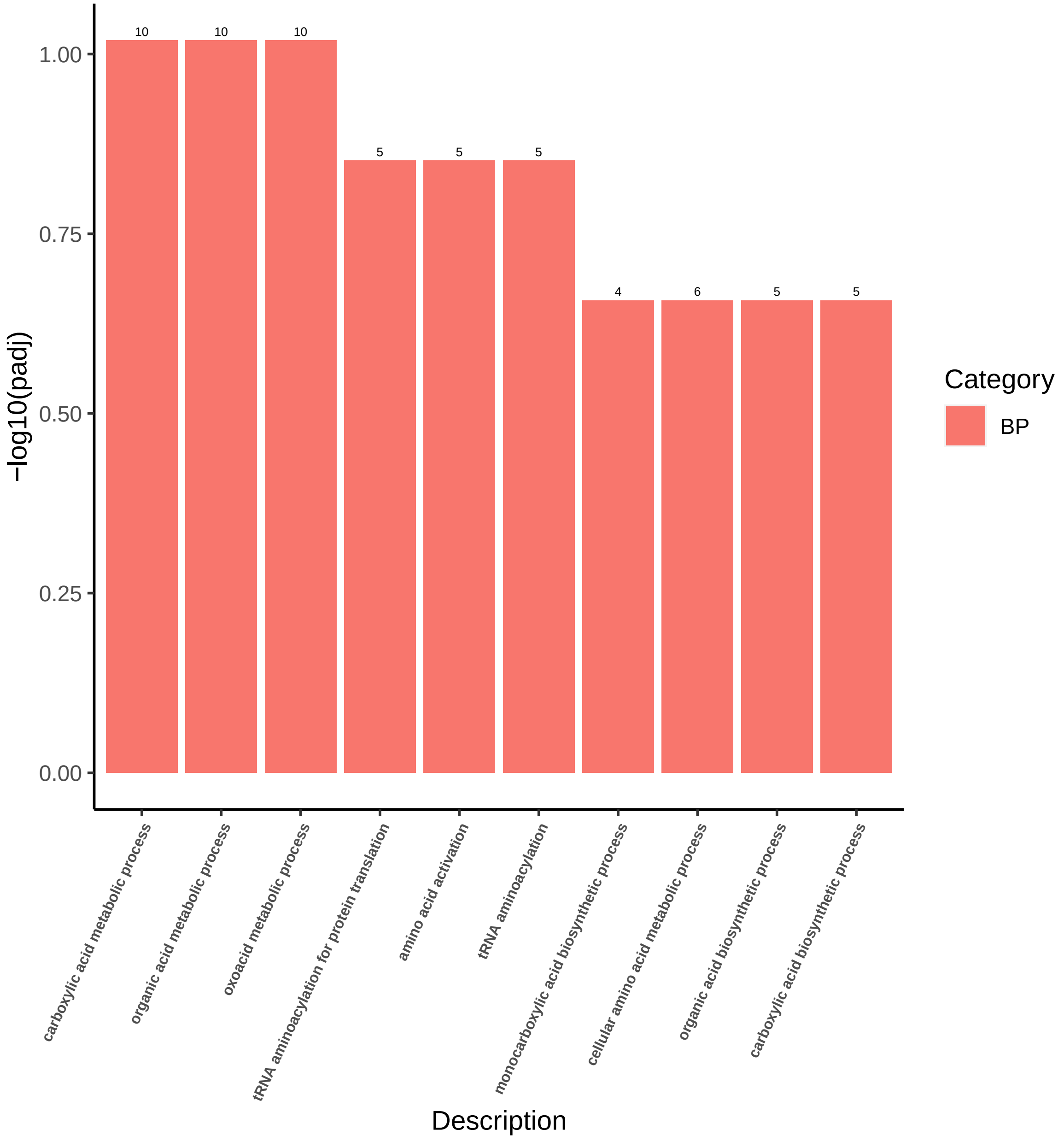

10
10
10
5
5
5
4
6
5
5
1.00
0.75
0.50
0.25
0.00
−log10(padj)
carboxylic acid metabolic process
organic acid metabolic process
oxoacid metabolic process
tRNA aminoacylation for protein translation
amino acid activation
tRNA aminoacylation
monocarboxylic acid biosynthetic process
cellular amino acid metabolic process
organic acid biosynthetic process
carboxylic acid biosynthetic process
Description
Category
BP
